# Adult height in girls with idiopathic central precocious puberty treated with triptorelin

**DOI:** 10.3389/fendo.2024.1498726

**Published:** 2024-12-05

**Authors:** Raquel Corripio, Leandro Soriano-Guillén, Francisco-Javier Herrero, Lidia Castro-Feijoó, Aránzazu Escribano, Paula Sol-Ventura, Rafael Espino, Amaia Vela, José-Ignacio Labarta, The Spanish PUBERE Group, Ignacio Díez López, Jesús Argente

**Affiliations:** ^1^ Pediatric Endocrine Department, Hospital de Sabadell, Corporació Parc Taulí, Institut Universitari Parc Taulí, Universitat Autònoma de Barcelona, Sabadell, Spain; ^2^ Pediatric Endocrinology Unit, Institute of Biomedical Research-Fundación Jiménez Díaz, Universidad Autónoma de Madrid, Madrid, Spain; ^3^ Pediatric Endocrinology Unit Hospital Sant Jaume de Calella, Corporació de Salut del Maresme i La Selva, Calella, Spain; ^4^ Pediatric Endocrinology, Growth, and Adolescence Unit, Hospital Clínico Universitario Santiago de Compostela, Universidad de Santiago de Compostela, Santiago de Compostela, Spain; ^5^ Pediatric Endocrinology Unit, Hospital Clínico Universitario Virgen de la Arrixaca, Murcia, Spain; ^6^ Pediatric Endocrinology Unit, Hospital Germans Tries i Pujol, Badalona, Spain; ^7^ Pediatric Endocrinology Unit, Hospital Valme, Valme, Spain; ^8^ Pediatric Endocrine, Hospital Universitario Cruces, Bilbao, Spain; ^9^ Pediatric Endocrinology Unit, Hospital Infantil Universitario Miguel Servet, Zaragoza, Spain; ^10^ Department of Pediatrics, Hospital Niño Jesús, Universidad Autónoma de Madrid, Madrid, Spain

**Keywords:** CPP, central precocious puberty, GnRHa, adult height, target height, GnRH agonists, idiopathic central precious puberty, girls

## Abstract

**Objective:**

Idiopathic central precocious puberty (CPP) precipitates epiphyseal fusion of growth plates in long bones, leading to reduced adult stature. Gonadotropin-releasing hormone analogues (GnRHa) are the treatment of choice for idiopathic CPP, but their benefit on height gain is unclear. We aimed to elucidate the effects of GnRHa treatment on adult height in girls with idiopathic CPP.

**Design:**

This prospective observational descriptive study analyzed data of girls with idiopathic CPP diagnosed at 55 centers in Spain between January 1, 1998 and December 31, 2012 included in the Spanish Society for Pediatric Endocrinology’s national registry.

**Methods:**

We included girls with idiopathic CPP (thelarche < 8 years, positive LHRH stimulation test, bone age > 1 year older than chronological age, and normal brain imaging) treated with triptorelin (3.75 mg monthly, adjusted according to LHRH test results and clinical findings). We assessed weight, height, BMI, and secondary sexual characteristics every 6 months and bone age every 12 months until adult height (AH) was attained. The primary outcome was the difference between AH and target height (TH).

**Results:**

A total of 465 girls (18.90% adopted) were included; we analyzed data recorded at treatment end in 358 girls and at AH in 216. Mean difference between AH and TH was -1.5 (95%CI: -2.56− -0.45) cm and between AH and PAH 2,57 (95%CI:-3.56− -1.58) cm.

**Conclusions:**

GnRHa treatment helps preserve genetic growth potential in girls with idiopathic CPP.

## Introduction

During puberty, reactivation of the hypothalamic-pituitary-gonadal axis leads to many physical, hormonal, and psychological changes (1, 2). This process is influenced by genetic, environmental, ethnic, metabolic, economic, and geographic factors (3–11). In girls, precocious puberty is defined as the development of secondary sex characteristics (Tanner stage 2 breast development) before the age of 8 years or menarche before the age of 9 years (12–14). Precocious puberty occurs in between 1:5,000 and 1:10,000 children, being 10 to 25 times more common in girls than in boys. Precocious puberty is classified in two categories according to its pathophysiology (15–17): peripheral precocious puberty, which is gonadotropin-independent, and CPP, which is gonadotropin-dependent. Most cases of precocious puberty are idiopathic CPP, which accounts for about 85% of all cases in girls and 65% of those in boys (3, 18).

Premature sex steroid hormone secretion in precocious puberty not only advances the progression of secondary sex characteristics, but also increases growth velocity, resulting in early fusion of epiphyseal growth plates in long bones and sometimes leads to short adult stature for the child’s genetic potential, which can also cause psychosocial maladjustment (19, 20).

Gonadotropin hormone-releasing hormone agonists (GnRHa) have been the treatment of choice for children with CPP since the 1980s (21–24). Administered chronically, GnRHa suppress the production of sex hormones, reducing growth velocity and giving the long bones more time to lengthen before epiphyseal fusion. As a result, bone age is progressively normalized, and linear growth continues, enabling children with CPP to achieve adult heights in line with genetic height potential (20, 25, 26). The main aims of GnRHa treatment are to interrupt sexual maturation until pubertal age, stabilize secondary sex characteristics, restore genetic height potential by delaying skeletal maturation, and prevent psychological problems (12, 27). The preferred route of administration is via a subcutaneous depot administered every month or every 3 months, because this approach ensures adherence (28); long-acting subdermal implants are also available (29–31). GnRHa are generally well tolerated; the most common adverse effects are local related to its injection (eg, allergic reactions or sterile abscesses), although headache, abdominal pain, vaginal bleeding after the first dose, and vasomotor symptoms have also been reported, as well as anaphylaxis, which is extremely rare (12, 27, 32). Moreover, GnRHa has also been associated with changes in body mass index (BMI) and an increased prevalence of polycystic ovary syndrome, although the evidence for these effects is inconclusive (33, 34).

The evidence, which is abundant in girls but sparse in boys, seems to demonstrate that GnRHa has favorable effects on longitudinal growth, although there is no consensus on the net gain in height resulting from GnRHa treatment, its effects on BMI, or the best time to discontinue treatment. Large prospective studies and randomized control trials are lacking, and much of the evidence is from studies with few subjects using historical cohorts reported decades before as controls and various other methodological limitations (12, 20, 26). Thus, the real height gain from GnRHa treatment remains to be determined.

In this study we aimed to define the effects of GnRHa treatment on AH in girls with idiopathic CPP. To this end, we report descriptive variables related to treatment, and we analyzed differences between and TH or predicted adult height (PAH) (in adopted girls whose genetical potential is unknown) and between AH and height at the discontinuation of GnRHa. We also compared height and other measurements against the general population of girls in Spain.

## Materials and methods

### Design and setting

This prospective observational descriptive study analyzed data of girls with idiopathic CPP diagnosed at 55 centers in Spain between January 1, 1998 and December 31, 2012 included in the Spanish Society for Pediatric Endocrinology’s national registry ( *www.seep.es/pubere* ) (3).

### Patients

We included girls with idiopathic CPP (defined as thelarche before 8 years of age, a luteinizing hormone (LH) peak > 7 U/L in a LH-releasing hormone (LHRH) stimulation test (100µg/m^2^), a difference between bone age and chronological age > 1 year, and normal cranial image findings (3)] treated with triptorelin (a GnRH agonist) administered at a monthly dose of 3.75 mg, adjusted when necessary, according to LHRH test results and clinical findings. Chemiluminiscence or Immunochemiluminometric (ICMA) was used for LH, FSH and sex steroids.

Follow-up consisted of examinations recording weight, height, BMI, and secondary sexual characters every 6 months and evaluating bone age every 12 months until AH was attained.

### Variables

Height-related variables are expressed in centimeters, weight in kg, and BMI in kg/m^2^; these variables are reported together with standard deviation scores (SDS) from the age- and sex-adjusted values for the general population in Spain (35). Bone age was assessed by comparing X-ray findings for the left hand with standard values, following the Greulich and Pile method (36); bone age and chronological age are reported in years. The duration of treatment and the time from the discontinuation of treatment to menarche are expressed in months. Secondary sex characteristics (eg, breast development) evaluated at physical examination are reported using Tanner pubertal staging (23).

### Outcomes

The primary outcome was the difference between AH and TH in girls whose biological parents’ height was known and between AH and PAH. Secondary outcomes were the difference between AH and height at the discontinuation of GnRHa as well as standard deviation scores for height and other measurements against the general population of girls in Spain.

### Definitions

*Target Height:* expected adult height estimated from factors such as genetics, parental heights, and sex. One common method for calculating TH is the mid-parental height method, based on the heights of the individual’s biological parents; for girls, the most common formula is:


TH=(father’s height + mother’s height-13)/2

*Adult height:* the height of an individual at their final adult stature, typically attained during late adolescence or early adulthood when the growth rate has stopped, indicating that they are at the end of their growth phase.

*Predicted adult height*: the height an individual is expected to attain based on their current chronological age and the degree of skeletal maturation indicated by their bone age. To determine this variable, we used the Bayley-Pinneau method (36), calculating the skeletal maturity ratio by dividing the child’s bone age by their chronological age and referring to reference tables (advanced column) to estimate the child’s eventual adult height. The updated version of this method has been in use since 1952 and has an IC95% of ± 6 cm (37).

### Statistical analysis

We aimed to achieve the largest possible sample, so no predetermined sample size was calculated. We report continuous variables as means with their 95% confidence intervals and categorical variables as frequencies and percentages.

We used a mixed regression model for repeated measures with AH as the independent variable and chronological and bone age at diagnosis, BMI-SDS, TH or PAH, and treatment duration as explanatory variables. Significance was set at p<0.05.

We used SAS v9.4 (SAS Institute Inc., Cary, NC, USA) for all analyses.

### Ethics statement

The Spanish Society of Pediatric Endocrinology’s clinical research ethics committee approved the study. The study was carried out in accordance with the principles laid out in the Helsinki Declaration, and patient anonymity was guaranteed according to Spanish law and European directives. Given the known benefits of GnRHa treatment, no control group of untreated patients was constituted. The study required no funding.

## Results

### Study population

**Figure 1:**
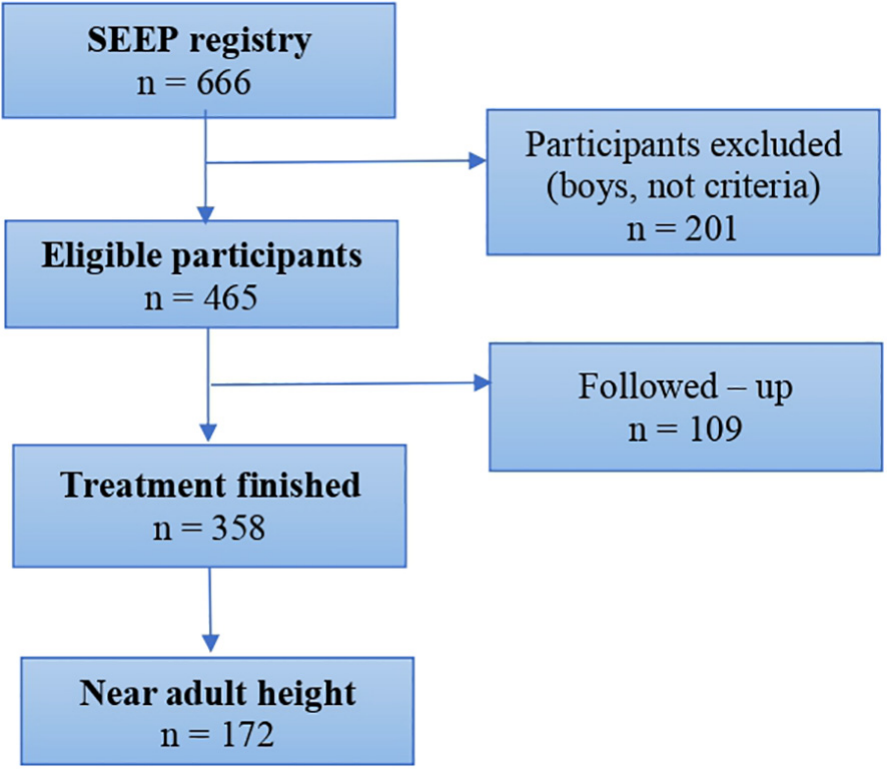
Study flowchart.

Of the 618 girls diagnosed with precocious puberty included in the SEEP registry, 465 fulfilled the inclusion criteria. Of these, 358 had finished treatment and 216 had reached AH (**Figure 1**). **Table 1** summarizes baseline characteristics of the included patients at diagnosis. At diagnosis, mean chronological age was 7.13 (95% CI:7.03−7.24) years and mean bone age was 9.24 (95% CI:9.11−9.37) years; the mean difference between chronological age and bone age was -2.09 (95% CI: 2.01-2.18 years).

**Table 1:** Baseline characteristics at diagnosis.

Baseline characteristics		N	Percentage	Mean	95% CI
Adopted	Adopted	88	18.92%		
	Not adopted	377	81.08%		
Immigration	Immigrant	58	12.47%		
	Non-immigrant	407	87.53%		
Residence	Urban	320	68.82%		
	Rural	145	31.18%		
Family background	Yes	85	20.33%		
	No	255	61.00%		
	Not registered	78	18.66%		
Tanner Stage	Stage II	296	64.35%		
	Stage III	154	33.48%		
	Stage IV	9	1.96%		
	Stage V	1	0.22%		
Maternal height (cm)		372		159.90	159.24−160.55
Paternal height (cm)		372		172.00	171.25−172.75
Mother’s menarche		350		11.81	11.63−11.98
Length at birth (cm)		292		48.86	48.51−49.20
Weight at birth (g)		373		2976.68	2916.15−3037.19
TH (cm)		370		159.45	158.91−159.98
TH SDS (cm)		370		-0.31	-0.41− (-0.22)
Chronological age (years)		463		7.13	7.03−7.24
Bone age (years)		461		9.24	9.11−9.37
Bone age – chronological age (years)		461		2.09	2.01−2.18
Height (cm)		461		127.80	126.95−128.64
Height (SDS)		461		1.51	1.38−1.63
PAH (cm)		435		159.33	158.49−160.18
BMI		461		17.79	17.57−18.00
BMI (SDS)		461		0.53	0.42−0.63
LH peak (U/L)		461		21.61	20.06−23.16

SDS, standard deviation score, based on age-adjusted values for the general population of girls in Spain; BMI, body mass index; LH, luteinizing hormone; PAH, predicted adult height; TH, target height.

### Treatment and outcome variables

**Table 2** reports treatment and outcome variables. Mean treatment duration was 2.57 (95% CI:2.43−2.71) years. At treatment end, mean chronological age was 10.03 (95% CI:9.93−10.13) years, and mean bone age was 11.67 (95%CI:11.57−11.76) years. Mean age at menarche was 11.23 (95%CI:10.5−11.92) years, and mean time between end of treatment and menarche was 14 (95%CI: 12.77−15.22) months.

**Table 2:** Treatment and outcomes.

Outcome	N	Mean	95%CI
Treatment duration (years)	355	2.57	2.44−2.72
CA at treatment end (years)	358	10.03	9.93−10.14
BA at treatment end (years)	303	11.67	11.58−11.76
BMI at treatment end (SDS)	357	1.06	0.93−1.20
Height at treatment end (cm)	357	144.7	143.94−145.46
Height at treatment end of (SDS)	357	1.31	1.18−1.442
Age at menarche (years)	272	11.23	11.10–11.368
Age at AH	216	13.54	13.30-13.77
Time from treatment end to menarche (months)	271	14.00	12.76−15.23
AH (cm)	216	157.3	156.3−158.29
AH (SDS)	216	0.57	0.384−0.749
AH – TH (cm)	216	-1.51	-2.56− -0.45
AH – TH (SDS)	216	0.89	0.72−1.06
AH – PAH (cm)	207	-2.57	-3.56− -1.58
AH - height at treatment end (cm)	209	12.08	11.45−12.7
AH - height at treatment end (SDS)	209	-0.76	-0.91 – -0.61
BMI at AH (SDS)	201	0.93	0.74−1.11

CA, chronological age; BA, bone age; BMI, body mass index; AH, adult height; TH, target height; PAH, predicted adult height.

From the discontinuation of treatment to adult height, the mean increase in height was 12.08 (95% CI:11.45−12.70) cm, that represents a mean increase in SDS height of -0.76 (95% CI: -0.91− -0.61) (n=216).

**Figure 2:**
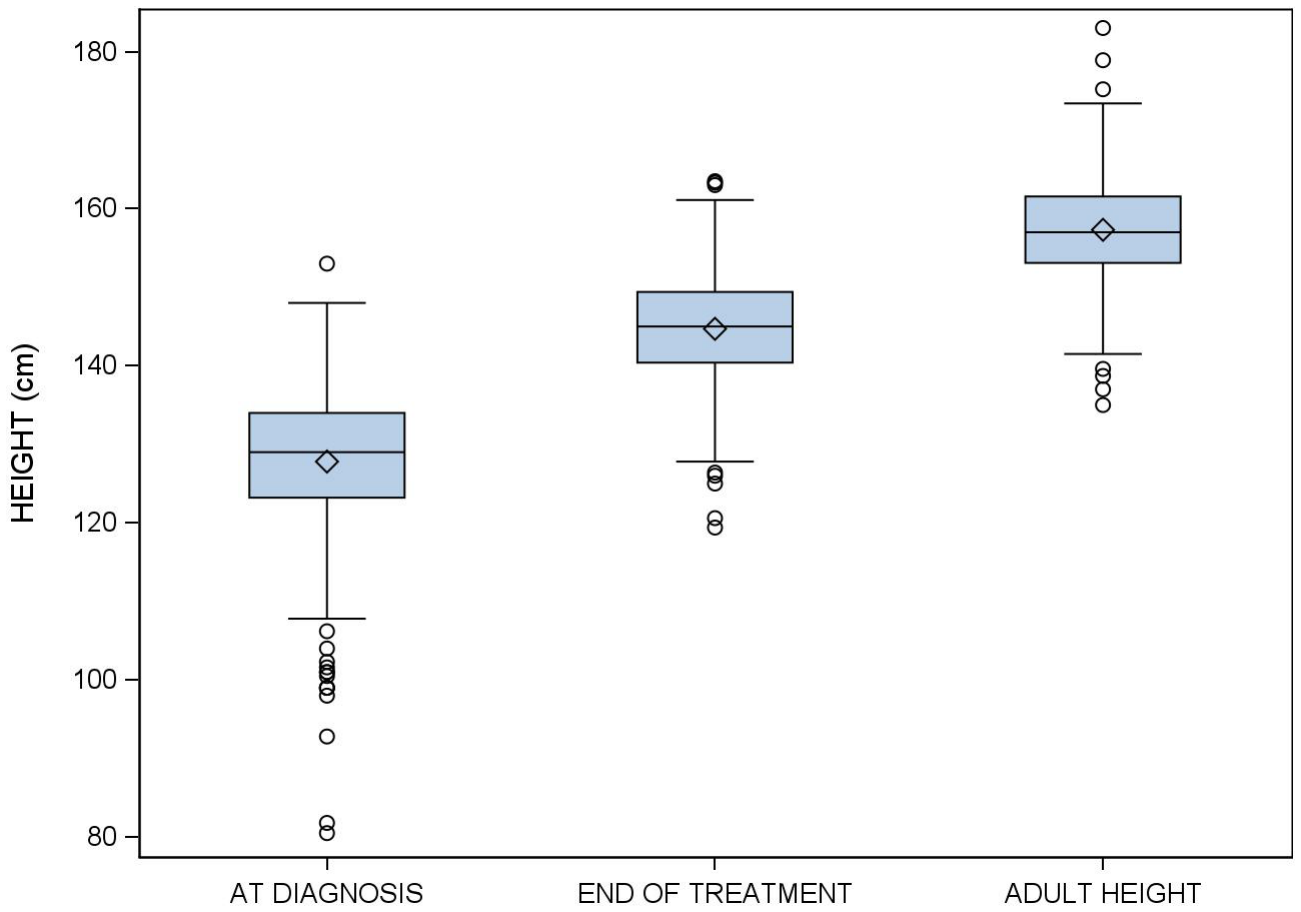
Evolution of height in girls with idiopathic central precocious puberty treated with triptorelin from diagnosis to adult height.

**Figure 3:**
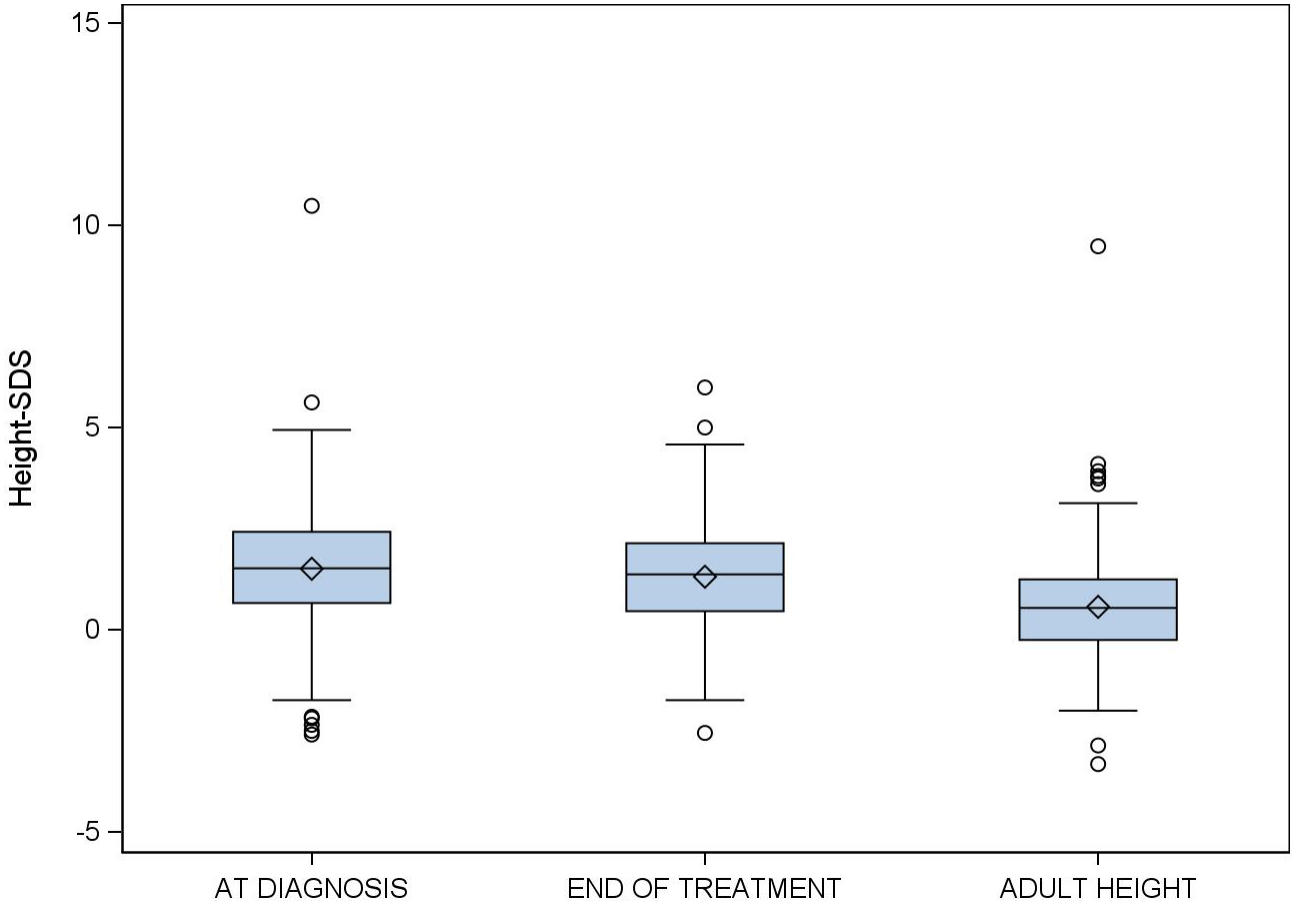
Evolution of standard deviation scores for height in girls with idiopathic central precocious puberty treated with triptorelin from diagnosis to adult height.

TH was attained in 71 (41%) patients. In the 102 (59%) patients who failed to attain TH, the mean difference between the TH and AH was 1.55 (95% CI:0.45−2.56 cm). **Figures 2**, **3** show changes in height from diagnosis to adult height.

**Figure 4:**
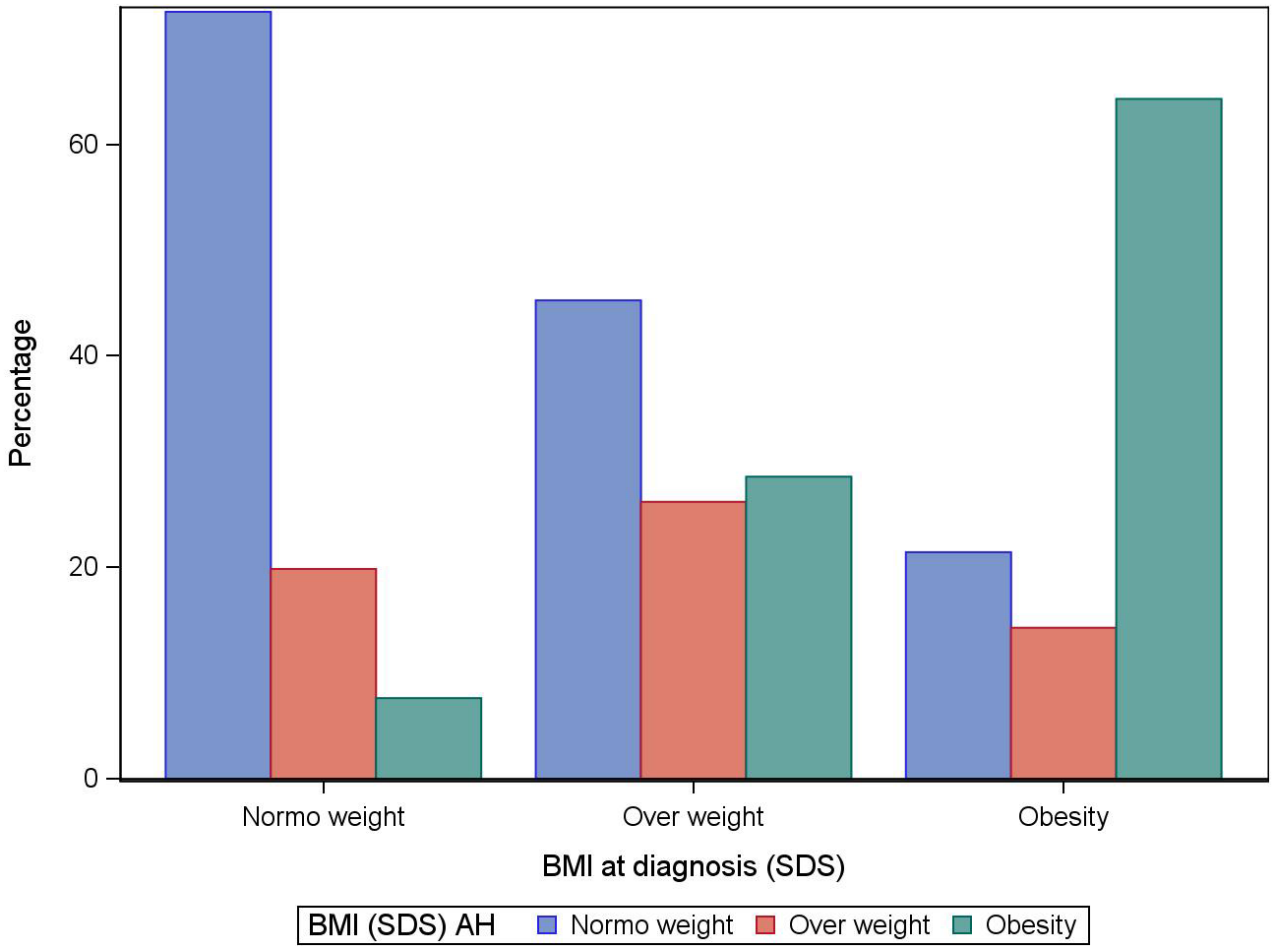
Standard deviation scores for body mass index (BMI) at adult height according to BMI at diagnosis of idiopathic central precocious puberty in girls.

BMI-SDS was 0.53 (95% CI:0.42−0.63) at diagnosis (n=461), 1.06 (95% CI:0.92−1.19) end of treatment (n=357), and 0.92 (95% CI:0.74−1.11) at AH (n=201) (p<0.05). Of the girls within the normal weight range at diagnosis, 72.5% remained in this range until adult height, 19.9% became overweight, and 7.6% became obese. Of the girls who were obese at diagnosis, 21.4% were within the normal weight range at adult height, 64.3% remained obese, and 14.3% were overweight. Of the girls who were overweight at diagnosis, 45% were within the normal weight range at adult height, 26.2% remained overweight, and 28.6% had become obese. The difference between BMI at AH and BMI at diagnosis was 0.43 SDS (95%CI:0.25−0.60; p<0.05) in the girls who were within the normal weight range at diagnosis. No significant differences in BMI between these two timepoints were observed in girls who were obese or overweight at diagnosis (**Figure 4**).

**Table 3** reports the results of the mixed regression model to predict adult height. Of the variables initially included, only BMI-SDS did not reach statistical significance. For every increase of one year in chronological age, AH increased 0.32 SDS; for every increase of one SDS in TH, AH increased 0.15 SDS; and for every increase of one SDS in height at diagnosis, AH increased 0.76 SDS. By contrast, for every increase of one year in bone age AH decreased 0.23 SDS, and for every increase of one year in treatment duration, AH decreased 0.09 SDS. Adverse effects of triptorelin were observed in 17 girls (3%). The most common adverse effects were spotting after the first dose (1.2%, n=6) and headache (0.6%, n= 3).

**Table 3:** Variables explaining adult height in the regression model.

Effect	Estimate	Standard Error	DF	t Value	Pr > |t|
Chronological age at diagnosis	0.3202	0.05863	1085	5.46	<0.0001
Bone age at diagnosis	-0.2269	0.04073	1085	-5.57	<0.0001
Target height (SDS)	0.1501	0.03978	1085	3.77	0.0002
Height at diagnosis (SDS)	0.7551	0.03102	1085	24.34	<.0001
Treatment duration (months)	-0.08980	0.03946	1085	-2.28	0.0230

SDS, standard deviation score with respect to the age-adjusted mean for the general population of Spanish girls.

## Discussion

Our large cohort of girls with idiopathic CPP has provided valuable information about GnRHa treatment. Although treatment of CPP with GnRHa is well established, the extent to which it can help correct short stature resulting from early fusion of the epiphyseal growth plates in long bones remained to be determined. For this reason, we sought to elucidate the effect of GnRHa treatment on AH by comparing this variable with TH. Except in adopted girls in whom it was impossible to calculate TH, we used TH rather than PAH to analyze the effects on AH to avoid the overestimation of PAH that commonly occurs in CPP, especially in patients with markedly advanced skeletal maturation.

Our results support the hypothesis that GnRHa has beneficial effects on growth. The TH was attained in 40% of patients, and the height attained by patients who failed to reach their targets was within the 5 cm interval indicated by the PAH. These findings are in line with those of other studies (20). In a review of the available data on GnRHa treatment for idiopathic CPP, Bereket (26) found that mean AH was 1 cm shorter than TH and concluded that GnRHa may not be capable of restoring full genetic height potential when started after a certain critical point in bone development. However, it could be argued that a difference of 1 cm is not clinically relevant, and this difference could be related to many different factors.

We found a significant difference between SDS for height at diagnosis, height at treatment end, and adult height. Historical series of untreated patients reported an average AH of 152 cm in girls (18). AH in our treated patients is higher (157.3 cm). Moreover, we observed further increases in height between GnRHa withdrawal and adult height, highlighting the importance of stopping the therapy at a specific time, giving bones enough time to continue growing. Along these lines, in addition to TH and chronological age, bone age, and height at diagnosis, our regression model identified treatment duration as a fair predictor of adult height, highlighting the importance of stopping treatment appropriately. This could explain why we stopped treatment earlier than other researchers and why our treatment duration is slightly shorter (38–40). Since height SD at diagnosis is a strong predictor of adult height, it should be considered that girls who are much taller than average at diagnosis may not require treatment if achieving normal adult height is the main consideration.

We analyzed BMI because CPP has been associated with overweight (41, 42), and historical cohorts and controlled clinical trials have found increases in BMI during GnRHa therapy, although BMI usually normalized after therapy was discontinued (34). In our cohort, we observed that BMI at diagnosis was 0.53 SD higher than in the age-adjusted population of Spanish girls; however, after AH was attained, BMI-SDI had increased only in patients whose weight was in the normal range at diagnosis. We speculate that patients that are overweight or obese receive stronger recommendations to diet and exercise and clinicians treating girls with CPP should point out the importance of these recommendations to all patients, regardless of their baseline BMI.

Our study has several limitations. As a control group of untreated patients would have been unethical, we can only compare our findings with historical cohorts. Comparisons with historical cohorts should be interpreted cautiously, taking into account possible differences in baseline characteristics, study design and inclusion criteria, and sample sizes. Likewise, we included only girls; factors affecting adult stature in boys with CPP are likely to differ. As in any study, random errors may be present; however, our large sample helps reduce their importance. Like most studies, we relied on the difference between target-height and AH to determine the effects of GnRHa treatment on stature; however, the midparental method of target-height calculation assumes equal contributions from both parents, neglecting the possible impact of dominant genes from one or the other (26). Furthermore, this study is not using the target height range and it could be possible that the difference in the final adult height and the TH, while significant for those on treatment may still be within an acceptable range of the target height. Finally, being overweight at diagnosis and adoption are risk factors for developing CPP, and they can act as confounding factors (3, 12).

## Conclusions

To summarize, our results suggest that GnRHa therapy is helpful in reaching a AH close to the TH and PAH, and this is beneficial in preserving genetic growth potential. Discontinuing treatment at the appropriate time favors significant growth afterward.

## Data Availability

The raw data supporting the conclusions of this article will be made available by the authors, without undue reservation.

## Collaborators

Álava, Hospital de Txagorritxu, Díez López Ignacio. Albacete, Hospital General de Albacete, Ruiz Cano Rafael. Alicante, Hospital Comarcal de Orihuela, Pastor Rosado José. Islas Baleares, Son Dureta, de Sotto Esteban Diego. Barcelona, Hospital Universitario Sagrat Cor, Cabrinety Perez Nuria. Barcelona,Hospital Vall Hebron, Yeste Fernández Diego. Barcelona, Hospital Sant Joan de Déu, Suárez Ortega Larisa. Barcelona Hospital Parc Taulí. Barcelona, Hospital del Mar. Bonet Alcaina Mariona. Barcelona, Hospital de Nens de Barcelona, Martín Ramos Rosa. Barcelona, Hospital Sant Joan de Déu de Manresa, Martí Aromir Gertrudis. Barcelona, Mutua Terrassa, Tomasini Rosangela. Barcelona, Hospital de la Santa Creu i Sant Pau, Carreras González Gemma. Barcelona, Hospital General de Granollers, Borrás Pérez María Victoria. Barcelona, Consorci Hospitalari de Vic, Torrabías Rodas Meritxell. Barcelona, Hospital de Mataró, Roqueta Sureda Montserrat. Barcelona, Hospital Germans Trias i Pujol de Badalona, Murillo Valles Marta. Cáceres, Hospital San Pedro de Alcántara, Arroyo Díez Francisco Javier. Cáceres, Hospital de Cáceres, González de Buitrago Jesús. Cádiz, Hospital Universitario Puerta del Mar, Lechuga Campoy José Luis. Cádiz, Hospital Universitario Puerta del Mar, Lechuga Sancho Alfonso. Córdoba, Hospital Universitario Reina Sofía, Cañete Estrada Ramón. A Coruña, Hospital Clínico Univ. de Santiago de Compostela, Castro Feijóo Lidia. A Coruña, Hospital Clínico Univ.de Santiago de Compostela, Barreiro Conde Jesús. A Coruña, Hospital Clínico Univ. de Santiago de Compostela, Cabanas Rodríguez Paloma. Lleida, Hospital Universitari Arnau de Vilanova de Lleida, Bosch Muñoz Jordi. Lugo, Hospital Xeral Calde de Lugo, Cepedano Dans Alicia. Madrid, Hospital Universitario Niño Jesús, Muñoz Calvo Mª Teresa. Madrid, Hospital Infantil La Paz, Guerrero Fernández Julio. Madrid, Hospital Ramón y Cajal de Madrid, Yturriaga Matarranz Rafael. Madrid, Hospital Universitario Doce de Octubre, Gallego Gómez Elena. Madrid, Hospital Universitario Doce de Octubre, Sánchez del Pozo Jaime. Madrid, Hospital Ramón y Cajal, Ros Pérez Purificación. Madrid, Hospital de Alcalá de Henares, Ramírez Fernández Joaquín. Madrid, Hospital General Univ. Gregorio Marañón, Rodríguez Arnao Mª Dolores. Madrid, Hospital General Univ. Gregorio Marañón, Rodríguez Sánchez Amparo. Málaga, Hospital Materno Infantil López Siguero Juan Pedro. Málaga, Hospital Materno Infantil Carlos Haya, Martinez-Aedo Ollero Maria Jose. Murcia, Hospital Universitario Virgen de la Arrixaca, Gutiérrez Macias Antonio. Murcia, Hospital Universitario Virgen de la Arrixaca, Martos Tello José María. Navarra, Hospital Virgen del Camino, Chueca Guindulain María. Navarra, Clínica Universitaria de Navarra, Azcona Sanjulián Cristina. Ourense, Hospital MI–Complexo Hospitalario Cristal-Piñor Fariña, Guerrero Pablo. Asturias, Hospital San Agustín, Riaño Galán Isolina. Las Palmas, Hospital Universitario Materno- Infantil Las Palmas, Quinteiro González Sofía. Sta. Cruz de Tenerife, Hospital Nuestra Señora de Candelaria, Rial Rodríguez José Manuel. Sta. Cruz de Tenerife, Hospital Universitario de Canarias, González Díaz Juan Pedro. Sevilla, Hospital Universitario de Valme. Espino Aguilar Rafael. Sevilla, H. Universitario Virgen de Valme, López-Canti Luis. Tarragona, Hospital Joan XXIII, Monné Gelonch Raquel. Teruel, Hospital Obispo Polanco, Martín Calama Jesús. Valencia, Hospital General de Sagunto, Valencia, Blasco Gonzalez Lidia. Valencia, Hospital General Universitario de Valencia, Alcón SáezJosé Juan. Valencia, Hospital Clínico Universitario, Plasencia Couchoud Amparo. Vizcaya, Hospital de Cruces. Baracaldo. Castaño González Luis. Vizcaya, Hospital de Cruces. Baracaldo. Martul Tobío Pedro. Vizcaya, Hospital de Cruces. Baracaldo. Rica Etxebarría Itxaso. Vizcaya Hospital de Basurto, Fernández Ramos Concepción. Zaragoza, Hospital Miguel Servet de Zaragoza. Lou Francés Gracia Mª. Zaragoza, Hospital General de la Defensa, Ruiz-Echarri Zalaya Manuel. Zaragoza, Hospital Clínico Universitario, Bueno Lozano Gloria.
